# Policy Evolution and Lessons Learned from China’s Efforts to Eliminate Leprosy

**DOI:** 10.1007/s44197-024-00188-7

**Published:** 2024-02-01

**Authors:** Ying Liu

**Affiliations:** School of Marxism, Fujian Normal University, Fuzhou, 350117 Fujian China

**Keywords:** Leprosy, Policies, Lessons, Elimination

## Abstract

The prevention and treatment of leprosy is a public health and social issue of global concern. China has become the first country in the world to put forward a proposal on the elimination of the harm caused by leprosy. This paper briefly introduces the status of the spread of leprosy in China, and systematically reviews the evolution of policies and measures at different stages of the disease in China, from the serious epidemic of leprosy to the control of the infection, to the basic elimination, and to the elimination of the hazards. On this basis, five main lessons learned from the control and elimination of leprosy in China were also summarized. These provide the basis for promoting the complete global elimination of leprosy and preventing its re-transmission, thereby benefiting all those who still suffer from the scourge of leprosy.

## Introduction

Leprosy is one of the chronic infectious diseases that seriously threaten and endanger human health. WHO has listed the disease as one of the 20 neglected infectious diseases [1]. Leprosy is classified as a category C infectious disease in China. According to the available research literature, leprosy had ravaged human beings for thousands of years and was one of the three major chronic infectious diseases in the world. People affected by leprosy existed in almost all countries and regions of the five continents [2], with some countries in Asia, Africa, and Latin America having the largest number of patients; while India, Egypt, and China were considered to be the three major sources of leprosy in the world. Historically, leprosy was once considered a shameful and incurable disease, containing meanings such as moral corruption, uncleanliness, susceptibility to infection and decay. Today, with the development of science and medical advances to make leprosy prevalence rate decreased significantly, but due to the leprosy bacteria have the particularity of being difficult to kill in the human body, and difficult to feed without leaving the human body, the medical community has not been studied to cure it, especially leprosy led to the disfigurement, disability, and treatment of the process of the occurrence of the adverse reaction of the serious drug and its resulting in the death of the hazards have not yet eliminated, resulting in leprosy is still a danger to human beings.

In China, leprosy was already recorded in oracle bone inscriptions in 1300 B.C. [3], and since then, leprosy has been a disease in China for more than 3000 years. Leprosy is said to have been imported from India [4], and in modern times, leprosy is still rampant in China, and the Chinese were considered a dangerous race for spreading leprosy around the world through immigration between the late nineteenth and mid-twentieth centuries [5]. It is estimated that there were a number of leprosy patients in China before 1949, but there are no exact statistics [6]. Leprosy was regarded as an "incurable disease", leaving a terrible impression in history and in people's memories—deformity, disfigurement, social ostracism, and abandonment, and widespread public fear of and discrimination against leprosy—and the resulting strong social reaction caused far more harm than the disease to both the patients and society. The strong social reaction so generated caused far more harm to the patient and society than the chronic disease itself.

After the founding of the People's Republic of China, this misfortune gradually became history, and through the unremitting efforts of the Chinese Government and the majority of professionals, always adhering to scientific prevention and treatment strategies and measures, supporting the production of leprosy patients and providing them with livelihood relief, the prevention and treatment of leprosy made remarkable achievements, and in 2000, the Ministry of Health of the People's Republic of China declared for the whole world that China's leprosy stock of patients had dropped to 2000 people, and the prevalence rate was less than one in 100,000 people, and leprosy had basically been eliminated from the country. After more than 70 years of struggle, China has finally become the first country in the world that has steadily eliminated the danger of leprosy. Therefore, a systematic review of the history of the fight against leprosy after the founding of New China from the perspective of the evolution of China's anti-leprosy policies and measures can provide basic experience for the promotion of the complete elimination of leprosy globally and the prevention of leprosy's re-infestation.

## Overview of the Spread of Leprosy in China

### Geographical Distribution

Leprosy in China is mainly distributed in the area south of 38°N latitude, especially in the south-east coast and Yangtze River basin, and also occurs sporadically in other provinces and regions; 86% of the cities had found patients, and the prevalence rate had been above 1/100,000 cities accounted for 69.1%, and the prevalence rate was above 1/100,000 cities accounted for 42.6%. In the 1950s and 1960s, the prevalence rate was 1/100,000 and the incidence rate was 1/100,000 or more in 19 provinces, including Guangdong, Yunnan, Hainan, Fujian, Jiangsu, Guangxi, Shanghai, Tibet, Guizhou, Shandong, Qinghai, Xinjiang, Jiangxi, Hunan, Sichuan, Shaanxi, Hubei, Gansu, Zhejiang, etc. Between 1949 and the end of 1997, the cumulative number of registered patients was 490,000 or more, with the cumulative number of registered patients in Guangdong, Shandong, Jiangsu, and Yunnan provinces being 50,000 or more [7]. By the end of 2000, there were still 250 leprosy-endemic cities with about 10% of leprosy prevalence, which had not reached the national target of basic leprosy elimination, 76% of which were in Yunnan, Guizhou, Sichuan, Tibet and Hunan [8].

According to the statistics of the "China Plan for the Elimination of Leprosy Hazard (2011–2020)", there were 278 cities in China with a prevalence rate of more than 1/100,000 by 2011, of which 46 had a prevalence rate of more than 1/100,000, mainly in the five provinces of Sichuan, Yunnan, Guizhou, Xizang and Hunan. In accordance with the prevalence and detection rates of leprosy in recent years, the "China Plan for the Elimination of Leprosy Hazard (2011–2020)" divided the provinces into three types of areas: Type I areas: Jiangxi, Hunan, Guangxi, Hainan, Sichuan, Chongqing, Guizhou, Yunnan, and Tibet; Type II areas: Jiangsu, Zhejiang, Anhui, Fujian, Shandong, Hubei, Guangdong, Shaanxi, Gansu, and Xinjiang; and Type III areas: Beijing, Tianjin, Hebei, Shanxi, Inner Mongolia, Liaoning, Jilin, Heilongjiang, Shanghai, Henan, Qinghai, and Ningxia. At present, leprosy is in a very low prevalence state in China [9]. It is mainly distributed in Yunnan, Guizhou, and Sichuan in the south-western region [10].

### Discoveries

Since the founding of New China in 1949, a cumulative total of more than 500,000 people have been found and treated for leprosy nationwide [10]. Among them, based on the nearly 600 million people in the 1953 national census, the number of new leprosy cases in the country was as high as 33,000 per year, with the highest incidence rate of 5.56/100,000 in 1958 [11]; from 1950 to 1987, the cumulative reduction in the number of leprosy cases in the country was about 400,000 (of which about 300,000 were cured), and by the end of 1987, there were only about 70,000 remaining cases of the current disease (the above statistics do not include Taiwan Province) [2]. During the period 1987–2000, the number of new cases of leprosy per year in the country declined from 4326 to 1603 cases [12]. In 1987, China introduced multi-drug therapy (MDT), and by the end of 1997, out of the cumulative total of more than 490,000 registered patients, more than 380,000 had been cured, of which 320,000 had been cured by monotherapy, 56,000 had been cured by MDT, and, excluding those reduced for other reasons, there are still 4045 patients in need of chemotherapy, with a prevalence rate of 0.033/10,000 [7]. By the end of 1999, there were still 6375 cases of patients with existing disease in China, accounting for 0.51/100,000 of the total population of China, and 89.8% of the cities in China had a prevalence rate of leprosy ≦ 0.1/100,000, and there were also 6.6% of the cities > 0.1/100,000, 1.7% of the cities > 0.5/100,000 and 1.9% of the cities > 1.0/100,000; according to the number of cases in need of treatment, there were still 19 cities (about 0.75% of the total) with prevalence > 1.0/10,000 (WHO target for leprosy elimination) [13]. The coverage of MDT approached 100% around 2000. The number of active leprosy patients decreased by 95.5% between 1987 and 2004 [14]. The annual detection rate of leprosy decreased from 5.56/100,000 in 1958 to 0.10/100,000 in 2010 [15]. From 2004 to 2021, in China (excluding Hong Kong, Macao Special Administrative Region and Taiwan), the number of incidence and incidence rate are listed in Table 1 [16].

**Table 1:** The number of incidence and incidence rate in China from 2004 to 2021

Year	2004	2005	2006	2007	2008	2009	2010	2011	2012	2013	2014	2015	2016	2017	2018	2019	2020	2021
Incidence	203	296	292	367	395	42	381	352	430	402	349	344	284	301	225	233	200	180
Incidence rate(/100,000)	0.0156	0.0228	0.0223	0.0279	0.0299	0.0319	0.0285	0.0263	0.0319	0.0297	0.0258	0.0252	0.0207	0.0218	0.0162	0.0167	0.0142	0.0128

## Evolution of Policy Measures for the Prevention and Control of Leprosy in China

After the establishment of the People's Republic of China, a series of policies and measures to combat leprosy were formulated and implemented. It is described according to the following three stages.

### Control of Contagion Stage (1949–1980)

After the founding of New China in 1949, the Chinese Government attached great importance to leprosy prevention and treatment. Under the leadership of the Ministry of Health, governments at all levels and health authorities formulated policies, trained personnel, established prevention and control institutions, investigated and detected patients, and gave amphotericin treatment in a timely manner, so that this stage was also known as the amphotericin monotherapy stage [17]. At this stage, through the determination of prevention and treatment guidelines, prevention and treatment policies and measures, the establishment of professional prevention and treatment organizations and professional prevention and treatment teams, the large-scale investigation of epidemics and the centralized free treatment (aminosulfone monotherapy being the mainstay), a total of more than 310,000 cases of leprosy patients had been cured, and the transmission and prevalence of leprosy had been under obvious control. The development of leprosy control was adapted at every critical stage in political history, thus becoming an integral part of the initial nation-building process [18]. Specific policies and measures adopted at that stage are described below:

In June 1950, the Ministry of Health issued a “Circular on Precautions to be Taken in the Management of Leprosy”, which established the policy of "prevention as the mainstay", and the first National Conference on Health and Epidemiological Control and the second National Conference on Health Administration, held in 1951 and 1952, respectively, were devoted to discussing the issue of the prevention and control of leprosy and to the preparation of programs for the training of professional and technical personnel and the establishment of prevention and control institutions. In 1953, the National Symposium on the Prevention and Control of Leprosy was held, at which the method of "combining prevention and treatment" was discussed and put forward. In 1956, the "National Programmes for Agricultural Development for the period 1956–1967(Draft)" was issued, which clearly put forward the instruction of "actively combating" leprosy, which gave a strong impetus to the importance of leprosy prevention and control work in various places, and after that, the work of leprosy prevention and control was gradually put on track. In June 1957, the Ministry of Health convened the "First National Leprosy Prevention and Control Work Conference", which examined many important issues relating to the prevention and control of leprosy. In October of the same year, the “National Plan for Leprosy Prevention and Control” was issued, establishing the principle of "active prevention and control of infection". On the basis of the training of technical staff, the identification of epidemics and the establishment of prevention and treatment institutions, it proposed the steps and practices of "investigation, isolation and treatment at the same time", and required party and government leaders at all levels to place the prevention and treatment of leprosy on their work program as an important task. At that time, the vast majority of leprosy patients were still living in the private sector, and in March 1959, the Ministry of Health and the Ministry of the Interior convened the "National Field Conference on the Prevention and Control of Sexually Transmitted Diseases, Leprosy and Tinea Capitis", which called for leprosy to be "detected, isolated and treated as early as possible", and after this conference, the number of leprosy villages nationwide increased from 114 to more than 700 within one and a half years after the launch of the "Great Leap Forward" [19], and a large number of leprosy patients from all over the country moved to leprosy villages for isolation.

In 1963, at a leprosy conference held by the Chinese Medical Association, technical documents such as the Leprosy Treatment Programmes and the Standards for Clinical Cure of Leprosy were formulated, and it was decided to adopt the classification scheme of the Sixth International Conference on Leprosy, and to generally promote the five-pronged comprehensive prevention and control measures, namely, "disease detection, sheltering, treatment, management and research", throughout the country. “The National Conference on the Exchange of Experience in Leprosy Prevention and Control”, held in November 1971 and October 1972, respectively, established the principles and measures for leprosy prevention and control, clarified the direction and tasks, and gave timely impetus to leprosy prevention and control throughout the country; and in March 1975, the State Council and the Central Military Commission forwarded to the Ministry of Health, the Ministry of Public Security, the Ministry of Finance, the Ministry of Agriculture and Forestry, the Ministry of Commerce, and the Ministry of General Logistics the "Report on the Strengthening of Leprosy Prevention and Control and Leprosy Control", which was published by the Ministry of Health. In November 1980, the State Council approved and transmitted the Ministry of Health's "Several Recommendations on the Situation of Leprosy Prevention and Control", which guided and promoted the healthy development of leprosy prevention and control throughout the country.

From the late 1950s to the early 1980s, there were 62 leprosariums, 343 prevention and treatment stations, and 794 leprosy villages in China; there were more than 9000 prevention and treatment specialists and technicians; the national leprosy prevalence rate had been controlled to less than 1/10,000 [20], and at least 200,000 people had been sheltered in isolation. Since 1980, Mahaid has been actively introducing special combination drugs for Chinese leprosy patients, which can eliminate infectiousness in 1 month and achieve curative effect in 2 years. Mahaid also advocated for home treatment and called for the banning of leprosy villages in the media [21]. He advocated for the full implementation of the new home treatment strategy and no new leprosy villages [22]. By the early 1980s, more than 310,000 cases of leprosy had been cured with amphotericin monotherapy [23].

### Basic Elimination Stage (1981–2000)

In 1981, China had already met the World Health Organization's criterion of "basic elimination of leprosy" by keeping the prevalence rate below 1/10,000, but China did not stop there, but set itself a new target of a prevalence rate below 0.1/10,000. At that stage, by setting the goal of eliminating leprosy, promoting multi-drug therapy, strengthening preventive and curative measures, and realizing the four transformations, as well as strengthening international cooperation, by the end of the twentieth century, in accordance with China's standard of "basic elimination" (prevalence rate of ≤ 0.1/10,000), China had achieved the goal of "basic elimination of leprosy" on a national basis. Specific policies and measures are described below:

In November 1981, the Ministry of Health held the "Second National Conference on Leprosy Prevention and Control", which, based on the achievements, experience and current situation of leprosy prevention and control in China over the past 30 years, put forward the goal of "striving for the basic elimination of leprosy in China by the end of this century". The Conference proposed the goal of "striving for the basic eradication of leprosy in China by the end of this century", and requested that by the year 2000, more than 95% of the cities in the country should have a prevalence rate of less than 0.1/10,000 (calculated on the basis of the number of active patients), and that the average morbidity rate (or the discovery rate) in the last 5 years should be less than 0.5/100,000, while in the other cities the prevalence rate should be less than 0.5/10,000; in view of the fact that after 1981, a large number of leprosy patients had been cured, and that the incidence of leprosy was declining both in terms of current cases and in terms of new cases, the Conference decided abandon the new leprosarium, village shelter isolation of leprosy patients, the use of chemical isolation (planned and focused to carry out multi-drug therapy), strengthen the rehabilitation of medical and other major decisions and requirements. The Meeting laid a solid foundation for the control and elimination of leprosy.

In 1981, the WHO study group recommended the leprosy MDT program, which was promoted all over the world. In 1982, MDT was introduced in China. In March, the Ministry of Health issued the “Situation and Future Opinions on the Prevention and Control of Leprosy”, and revised and issued seven technical documents, such as the “National Regulations on the Administration of Leprosy”, “Prevention and Control and the Trial Programme of Multi-drug therapy for Leprosy”. They are in the nature of administrative regulations, which not only guide the management of leprosy prevention and control, but also guarantee the goal of basic leprosy elimination. In July 1984, the Ministry of Health issued the “Opinions on Strengthening the Prevention and Control of Leprosy”; in October, it decided to establish the China Leprosy Prevention and Control Research Centre; and in December, it issued the “National Leprosy Prevention and Control Regulations for the Period 1985–2000” [2]. In the meantime, all provinces, cities and autonomous regions where leprosy was prevalent had formulated long-term plans and recent arrangements for the prevention and control of leprosy, adjusted leprosy prevention and control institutions (mainly leprosariums and villages were closed down, suspended, merged and transferred), strengthened social prevention and control and publicity work, carried out MDT with plans and priorities, and accelerated the pace of prevention and control, and requested that the basic elimination of leprosy be achieved by phases in the form of counties and cities as a unit. “China Leprosy Prevention and Control Association, China Leprosy Welfare Foundation and China Leprosy Prevention and Control Research Centre inaugural meeting and China's first International Leprosy Academic Exchange Conference" was held, which was a bridge and link for the Chinese government to contact leprosy scientific and technological workers, and marked the transformation of leprosy prevention and control in China from a lone battle by the prevention and control professional team to the mobilization of social forces (mass organizations and various circles) and publicity work. From this year onwards, Chinese experts began to participate in international symposia and congresses, and China began to participate more actively in the WHO program [18]. We actively strengthened international and domestic horizontal ties with WHO and leprosy relief agencies and foundations in Japan, Belgium, Netherlands, Italy, the United States, the United Kingdom, Germany, and other countries, all of which sponsored leprosy prevention and treatment in China, and supported the prevention and treatment of leprosy by providing medicines and equipment (especially medicines urgently needed for the implementation of the MDT), means of transport, and funds for training, which facilitated the advancement of leprosy prevention and treatment work in China [2].

The National Symposium on MDT for Leprosy held in December 1986, after gaining experience through pilot projects, decided to adopt the MDT program designated by WHO, and requested that it be universally implemented in the whole country in 1987, as a key point of the comprehensive preventive and curative measures, and that "the MDT program has become one of the most important means for the elimination of leprosy globally and for the basic elimination of leprosy in China” [24]. The proposal "Towards the Elimination of Leprosy" put forward by 23 countries, initiated by China, was unanimously adopted by the 40th World Health Assembly in May 1987, which resolved that the elimination of leprosy should be an integral part of the goal of "Health Care for All by the Year 2000″. The Third National Conference on Leprosy Prevention and Control, held in November 1987, was an important step forward in China's leprosy prevention and control strategy and measures; the conference put forward the policy of accelerating and deepening the reform of leprosy prevention and control, and proposed four changes in preventive and control measures [7]: (i) change from single-drug treatment to multi-drug therapy (MDT) (ii) changing the inpatient isolation of leprosy patients to out-of-hospital social prevention and treatment; (iii) changing from pure treatment to a combination of treatment and rehabilitation; and (iv) changing from a lone professional team to mobilization of social forces for concerted action. The Conference identified Yunnan, Guizhou, Sichuan, and Tibet as the focus of prevention and treatment at the national level, and requested all provinces, municipalities, and autonomous regions to point out to them with a view to reaching the basic elimination target on schedule; for areas where the incidence and prevalence of leprosy had already declined significantly, it was requested that the basic elimination target be reached ahead of schedule. In terms of comprehensive prevention and treatment measures, emphasis was placed on MDT, rehabilitation and medical care and publicity.

In 1988, the prevention and control of leprosy throughout the country had progressed from basic control to basic elimination, and in January, the Ministry of Health revised the “National Regulations on the Administration of Leprosy Prevention and Control”, the “Standards for Combined Chemotherapy and Evaluation of Leprosy”, the “Rehabilitation and Medical Treatment of Leprosy Deformities and the Interim Measures for the Examination and Acceptance of the Basic Elimination of Leprosy (Interim)”, which were sent out for implementation in September of the same year [2]. In August 1993, the State issued the "Measures for the Examination and Acceptance of the Basic Elimination of Leprosy", which set new standards for the indicators of the basic elimination of leprosy: the prevalence rate is ≤ 0.1/100,000 inhabitants, average annual incidence rate for the past 5 years ≤ 0.5/100,000.

From 1987, when MDT was fully implemented nationwide, to the end of 1997, the cumulative number of people who received MDT reached 77,000, with the coverage rate above 97% since 1990, and the cumulative number of people who completed MDT was 64,000, with the expected completion rate above 95% [7]. From 1949 to the end of 1997, the cumulative number of registered patients was more than 490,000, and more than 380,000 were cured, of which 320,000 were cured by monotherapy and MDT cured 56,000, excluding those who were reduced for other reasons, there were still 4045 patients who needed chemotherapy, with a prevalence rate of 0.33/10,000 people. The number of patients with presenting symptoms has been greatly reduced through the full-scale promotion of MDT programs. Some statistics show that the coverage of MDT was close to 100% around the year 2000 [25].

In accordance with the WHO standard of "basic elimination of leprosy" (prevalence rate ≤ 1/10,000), China met the WHO requirement of keeping the prevalence rate below 1/10,000 in 1981; provinces met it in 1992; and at the cities reached the level of in 1997. In accordance with China's "basic eradication" standard (prevalence rate ≤ 0.1/10,000), by the end of 1997, 85% cities nationwide had reached the target of a prevalence rate of less than 0.1/10,000, calculated on the basis of active cases [7]. By the end of 1999, 98.1% cities nationwide had a prevalence rate of < 10/100,000 (WHO's indicator for the elimination of leprosy), which was in a low prevalence state, and 89.9% had a prevalence rate of 1/100,000 (one of China's indicators for the basic elimination of leprosy), which was in line with the requirement of "basic elimination” [26]. In 2000, the Ministry of Health announced to the world that the number of leprosy patients in China had dropped to about 2000, with a prevalence rate of less than 1/100,000, and that leprosy had been basically eliminated in the country [27].

### Hazard Elimination Stage (2001–present)

Entering the twenty-first century, leprosy was at a low prevalence stage in China, with a significant reduction in the number of new and existing cases of leprosy, and the promotion of integrated prevention and treatment measures continued, while focusing on issues such as leprosy deformities and discrimination, as China entered a new era of elimination of the harm caused by leprosy. During this period, the Government of China implemented the reform of integrated leprosy prevention and treatment, the Government of China implemented leprosy prevention and treatment projects, the Government of China invested in the renovation and construction of leprosariums (villages), and the 11 departments of the Government of China made commitments to the prevention and treatment of leprosy, among others. Specific policy measures are listed below:

In April 2001, the Government of China approved the “Outline of the Tenth Five-Year Plan (2001–2005) for the Cause of Persons with Disabilities in China”, which for the first time included "providing corrective surgery or assistive devices for the 120,000 existing persons with leprosy deformities and disabilities, so as to improve the quality of their lives" in the Tenth Five-Year Plan; the “Marriage Law of the People's Republic of China” canceled the provision prohibiting the marriage of leprosy patients; in the same year, the Ministry of Health issued the “National Plan for the Prevention and Treatment of Leprosy (2001–2005)”. In 2004, the central financial administration included the prevention and treatment of leprosy into the public health special program, and governments at all levels also gave stable financial inputs, and at present, the diagnosis and treatment of leprosy are free of charge in China [28]. In 2006, China put forward the concept of "relying on scientific and technological progress to promote the prevention and treatment of leprosy", and explored the immunogenetic mechanisms that caused the harm caused by leprosy; to promote the prevention and treatment process of leprosy, the Ministry of Health issued the "National Plan for Prevention and Treatment of Leprosy (2006–2010)” [29]. In June 2008, the Chinese Government signed “the resolution on the elimination of discrimination against persons affected by leprosy, persons cured of leprosy and their family members”, adopted by the United Nations Human Rights Council, and allowed persons affected by leprosy from outside China and their family members to be admitted to the program [30]. In 2010, the Government of China adopted the “Rules for the Implementation of the Law of the People's Republic of China on Sanitary and Quarantine at the State Border” and the “Implementation Law for the Administration of the Entry and Exit of Foreigners into the Programme”, removal of restrictions on the admission of foreigners suffering from infectious diseases such as leprosy [31].Since the “Implementation of the National Plan for Eliminating the Hazards of Leprosy (2011–2020)”, jointly issued by the Ministry of Health and 11 other departments in September 2011, China has made continuous progress in the prevention and treatment of leprosy, achieving the goal of controlling the prevalence rate of leprosy in more than 98% cities nationwide to less than 1/100,000, and the scope of leprosy epidemics has continued to be narrowed, and the intensity of epidemics has been further reduced [32]. On September 17, 2016, General Secretary Xi Jinping proposed in his congratulatory letter to the 19th International Leprosy Congress that "China will increase investment and safeguards, continue to work together with other countries around the world to actively promote leprosy progress and innovation, and facilitate the goal of eradicating leprosy to be achieved in China at an early date". In 2020, under the leadership of the Government of China, and after more than 70 years of struggle, China finally become the first country in the world to have steadily eliminated the harm caused by leprosy [27].

Since the establishment of the People's Republic of China, China has made increasing efforts to detect, treat and manage leprosy patients, especially in recent years, China has made steady progress in eliminating the risk of leprosy, with close to 98% of the cities nationwide reaching the target [33], and leprosy in China is now in a very low prevalence state. In the future, China will continue to promote comprehensive measures for the prevention and treatment of leprosy, and strive for the complete and thorough elimination of leprosy at an early date.

## Lessons Learned from the Control and Stable Elimination of Leprosy in China

In the fight against leprosy over the past 70 years, China has gone through a process from controlling the infection to basic elimination to elimination of the hazard, which is both a top-down "national action" and has obvious epidemiological characteristics and social factors. In sum, China's main experiences in controlling and steadily eliminating leprosy can be summarized as follows.

First, the Government attached full importance to it and took strong political action. After the founding of the People's Republic of China, China has always taken leprosy control as a governmental act and "national action", incorporating it into the overall situation of health and social development, with the Government at all levels, from the central to the local level, making unified arrangements, dividing up the work and making corresponding inputs, and providing free diagnosis and treatment for leprosy. The State successively formulated the “National Regulations on the Administration of Leprosy Prevention and Control”, the “Measures for the Evaluation and Acceptance of the Basic Elimination of Leprosy”, and the “National Leprosy Prevention and Control Plan”; all localities formulated their own regional control plans, and carried out their work in a systematic and systematic manner. Leading groups for leprosy prevention and control were set up in endemic areas, consisting of departments of health, finance, civil affairs and publicity, to work together to solve major problems in the work of the area. The Chinese Leprosy Prevention and Control Association, the China Leprosy Foundation, the China Leprosy Prevention and Control Research Centre and other leprosy institutions were being improved, and the State was investing more financial, material and scientific research resources in order to solve major problems in prevention and control work.

Second, there were sound leprosy control teams and grass-roots control networks. A combination of well-trained professional leprosy prevention and treatment teams with a spirit of professionalism and dedication, as well as a relatively sound grass-roots health-care network, has been established, especially in endemic areas, with a relatively complete leprosy prevention and treatment system, leading to the participation of the public in carrying out mass prevention and treatment. Provincial, municipal and county leprosy control teams were actively selected to participate in national training courses on leprosy prevention and treatment, deformity prevention and rehabilitation, and to provide training for medical personnel at all levels, using them as teaching staff. Professionals were selected and sent to participate in meetings for the exchange of experience in international cooperation projects on leprosy, so as to improve their ability to work in leprosy prevention and treatment. At the same time, medical and health institutions at all levels in the affected areas were actively organized to take on the task of preventing and controlling leprosy, and the majority of Chinese and Western medicine and health workers in the affected areas were mobilized to actively participate in leprosy prevention and treatment, organized to take on the task of investigating and discovering patients, as well as providing medical treatment and publicity and education to local patients with leprosy in the diaspora. Through public participation, the implementation of comprehensive measures for the prevention and treatment of leprosy was ensured.

Third, socio-economic development and progress. Although the contagiousness of leprosy is related to the host, the pathogen and the environment, the occurrence of leprosy is also often related to poor socio-economic conditions; the spread and prevalence of leprosy is an important sign of poverty and backwardness in a country, and the economy is the basis for determining the level of health, thus the prevalence of leprosy to a certain extent reflects the process of the spiritual civilization and material civilization of a country or region. The rapid socio-economic development after the founding of the People's Republic of China, especially after China's reform and opening up, has enabled continuous improvement in the standard of living (e.g., housing, diet, nutrition, etc.) and medical and health conditions of the population at large, and funds for leprosy prevention and treatment could be sufficiently invested, thus advancing the process of leprosy prevention and treatment.

Fourth, scientific prevention and control. In the course of leprosy prevention and treatment, China has carried out mass patriotic health campaigns, publicized the fact that "leprosy is preventable, curable and not to be feared", put forward the concept of "relying on scientific and technological progress to promote the prevention and treatment of leprosy", explored the immunogenetic mechanism that causes leprosy, and conducted large-scale filtering censuses and key clue surveys nationwide for a number of times. A number of large-scale nationwide filtering censuses and surveys of key clues were carried out, and a large number of epidemiological analyses and research results were produced accordingly, which were conducive to determining the types of leprosy patients in China, their geographic distribution, the number of leprosy patients and other important data, so that targeted prevention and treatment could be carried out. China's leprosy prevention and control workers also discovered the prevalence of leprosy susceptibility factors in human DNA, making early diagnosis of leprosy possible. China has also established the largest leprosy bank in the world, a biological resource bank, on the basis of which risk factors for the development of leprosy have been discovered through major cooperation at home and abroad, a prediction model for the risk of the development of leprosy has been constructed, a screening chip for high-risk individuals has been developed, and precise chemoprophylactic dosing for leprosy has been realized, contributing to the elimination of the primary hazards of leprosy, as well as the effective elimination of secondary hazards of leprosy.

Fifth, strengthening cooperation with the international community. China's leprosy prevention and treatment focuses on international integration, taking the approach of "going out and inviting in" to strengthen exchanges and cooperation with the United Nations World Health Organization (WHO) and international leprosy prevention and control professional bodies, while at the same time drawing on the new ideas and practices of leprosy prevention and treatment in foreign countries, and obtaining the strong support of international organizations, non-governmental organizations, and friendly people who have provided a lot of help in terms of funding, materials, and technology, thus accelerating the process of leprosy prevention and control in China.

## Conclusion

China was the first country in the world to propose the elimination of leprosy, and after more than 70 years of active prevention and treatment, the elimination of leprosy in China has made remarkable achievements, changing the destiny of hundreds of thousands of leprosy sufferers, and turning leprosy sufferers from "desperation for death" to "rescue and rebirth", which is a milestone in the history of the development of public health in China, leaving a glorious page in the history of China and the world, contributing to the creation of a leprosy-free world with Chinese programs and Chinese power. This has not only greatly reduced the total number of leprosy cases globally, but also strongly boosted the confidence of the international community in interrupting the spread of leprosy and achieving zero local cases and the complete elimination of the harm caused by leprosy. Leprosy, as a global health problem, still requires joint global efforts to ultimately achieve its complete elimination. We hope that by understanding China's historical experience for controlling and steadily eliminating the harms of leprosy, we can contribute to the global plan for the complete elimination of leprosy, and in particular to the advancement of the implementation of the WHO's Global “Leprosy Strategy for the period of 2021–2030”, and the realization of a world free of leprosy infections, stigma, and discrimination associated with leprosy.

## Data Availability

The datasets used and/or analyzed during the current study are available from the corresponding author on reasonable request.

## Abbreviations

B.C.: Before Christ
DNA: Deoxyribonucleic acid
MDT: Multi-drug therapy
WHO: World Health Organization
